# Sleep disorders: comparison of ICD-11 and ICD-10

**DOI:** 10.1007/s00115-025-01859-x

**Published:** 2025-09-18

**Authors:** Kai Spiegelhalder, Dieter Riemann

**Affiliations:** Department of Psychiatry and Psychotherapy, Medical Center—University of Freiburg, Faculty of Medicine, University of Freiburg, Hauptstraße 5, 79104 Freiburg, Germany

**Keywords:** Sleep-wake disorders, Insomnia disorder, Hypersomnias, Circadian rhythm sleep disorders, Parasomnias, Schlaf-Wach-Störungen, Insomnische Störungen, Hypersomnische Störungen, Zirkadiane Schlaf-Wach-Rhythmus-Störungen, Parasomnische Störungen

## Abstract

The current article reviews adjustments that were made to the classification of sleep disorders in the 11th revision of the International Statistical Classification of Diseases and Related Health Problems (ICD-11) in comparison to the 10th revision of the coding system (ICD-10). A new chapter on sleep-wake disorders was introduced as chapter 7 in ICD-11, removing the distinction in nonorganic and organic sleep disorders that was used in ICD-10. The rationale for this was the commonsense notion that clinicians and researchers have difficulties to identify the etiology of insomnia and to establish causality between insomnia and coexisting conditions. With respect to sleep disorders that were previously included in chapter V “Mental and behavioural disorders” of the ICD-10, the following important changes were made: the diagnosis of insomnia disorder can now be made as comorbid with other mental disorders or physical illnesses if the insomnia symptoms are a focus of independent clinical attention, non-restorative sleep alone without difficulties initiating or maintaining sleep is not sufficient anymore to diagnose insomnia disorder and new diagnostic categories have been created, including insufficient sleep syndrome and sleep-related eating disorder. Future research will show whether the adjustments in ICD-11 will help clinicians to make reliable and clinically useful diagnoses and whether this improves routine clinical care for sleep disorders.

## Abstract (de)

In der 11. Revision der Internationalen statistischen Klassifikation der Krankheiten und verwandter Gesundheitsprobleme (ICD-11) der Weltgesundheitsorganisation haben sich hinsichtlich der Schlafstörungen substanzielle Änderungen gegenüber der 10. Revision (ICD-10) ergeben. Vor allem werden die in der ICD-10 in verschiedenen Kapiteln genannten Schlafstörungen in der ICD-11 in einem Kapitel „Schlaf-Wach-Störungen“ zusammengefasst. Damit entfällt die Unterscheidung in nichtorganische und organische Schlafstörungen, die im klinischen Alltag einerseits schwierig zu treffen ist und andererseits geringe therapeutische Bedeutung hat. In Bezug auf Schlafstörungen, die vormals dem Kapitel V „Psychische und Verhaltensstörungen“ der ICD-10 zugeordnet waren, ergaben sich u. a. folgende wichtige Änderungen: Die insomnischen Störungen können nun komorbid zu anderen psychischen Störungen oder körperlichen Erkrankungen diagnostiziert werden, wenn sie einen Fokus der unabhängigen klinischen Aufmerksamkeit darstellen, das Kriterium nichterholsamer Schlaf ist als nächtliches Symptom ohne Ein- und Durchschlafstörungen nicht mehr ausreichend, um eine insomnische Störung diagnostizieren zu können, und für einige Krankheiten werden neue Diagnosen geschaffen, u. a. dem Schlafmangelsyndrom und der schlafbezogenen Essstörung. Zukünftige Forschung wird zeigen müssen, ob die Veränderungen in der ICD-11 zu einer reliablen und klinisch nützlichen Diagnostik von Schlafstörungen führen und insgesamt zu einer Verbesserung der Routineversorgung beitragen können.

In the 10th revision of the International Classification of Diseases and Related Health Problems (ICD-10), sleep disorders are listed both as “nonorganic sleep disorders” in Chap. V, “Mental and Behavioural Disorders” (F51.-), and as “sleep disorders” in Chap. VI, “Diseases of the Nervous System” (G47.-). Instead, in ICD-11, all sleep disorders are included in a separate chapter, Chap. 7 “Sleep–Wake Disorders.” This chapter encompasses insomnia disorders, hypersomnolence disorders, sleep-related breathing disorders, circadian rhythm sleep–wake disorders, sleep-related movement disorders, and parasomnia disorders.

The grouping of sleep–wake disorders into one chapter resembles the elimination of the terms “primary insomnia” and “secondary insomnia” in the fifth edition of the Diagnostic and Statistical Manual of Mental Disorders (DSM-5). The DSM‑5 category “insomnia disorder” can be diagnosed comorbidly with other mental disorder or physical illnesses. This conceptual shift reflected the recognition that, although insomnia disorder frequently co-occurs with other mental disorders or physical illnesses, a clear causal relationship cannot be established in most cases. Instead, insomnia often precedes the onset of comorbid disorders or persists beyond their successful treatment [2, 3].

Whether the introduction of the chapter “Sleep–Wake Disorders” in ICD-11 will lead to improved care for patients is difficult to predict. Many sleep medicine specialists regard the inclusion of this chapter as recognition of the clinical relevance of sleep disorders. Moreover, insomnia is more clearly recognized as a disorder in its own right in ICD-11 than it was in ICD-10. However, the reclassification of insomnia disorder and nightmare disorder as conditions no longer listed under mental and behavioral disorders may make it more difficult for patients to access appropriate care. This shift may result in fewer healthcare professionals considering themselves responsible or adequately trained to treat sleep disorders. For instance, clinicians in psychiatry, psychotherapy, psychosomatic medicine, and neurology may increasingly view their primary area of expertise as limited to conditions listed in the chapter “Mental, Behavioural or Neurodevelopmental Disorders,” and less so to diagnoses in other chapters of ICD-11.

In the chapter on sleep–wake disorders, several structural and content-related changes have been introduced, which are summarized in this article. The article focuses on diagnostic categories that include disorders previously classified under Chap. V, “Mental and Behavioural Disorders” in ICD-10. Accordingly, sleep-related breathing disorders, which are typically diagnosed and treated in the fields of pulmonology or otorhinolaryngology, are not within the scope of this article. A comparison of the relevant ICD-10 and ICD-11 diagnoses is provided in Table 1.

**Table 1:** Sleep disorders in ICD-10 and ICD-11

ICD-10	ICD-11
**Behavioral syndromes associated with physiological disturbances and physical factors**	**Sleep**–**wake disorders**
–	*Insomnia disorders*
Nonorganic insomnia (F51.0) Disorders of initiating and maintaining sleep (insomnias) (G47.0)	Chronic insomnia (7A00)
	Short-term insomnia (7A01)
Nonorganic sleep disorder, unspecified (F51.9) Sleep disorder, unspecified (G47.9)	Hyposomnia, unspecified (7A0Z)
–	*Hypersomnolence disorders*
Narcolepsy and cataplexy (G47.4)	Narcolepsy (7A20)
Idiopathic hypersomnia (G47.11) Nonorganic hypersomnia (F51.1)	Idiopathic hypersomnia (7A21)
Other sleep disorders (G47.8)	Kleine–Levin syndrome (7A22)
Hypersomnia due to a medical condition (G47.14)	Hypersomnia due to a medical condition (7A23)
/	Hypersomnia due to a medication or substance (7A24)
Nonorganic hypersomnia (F51.1)	Hypersomnia associated with a mental disorder (7A25)
Other nonorganic sleep disorders (F51.8)	Insufficient sleep syndrome (7A26)
Nonorganic sleep disorder, unspecified (F51.9) Sleep disorder, unspecified (G47.9)	Hypersomnolence disorders, unspecified (7A2Z)
–	*Circadian rhythm sleep*–*wake disorders*
Circadian rhythm sleep disorder, delayed sleep phase type (G47.21)	Delayed sleep–wake phase disorder (7A60)
Circadian rhythm sleep disorder, advanced sleep phase type (G47.22)	Advanced sleep–wake phase disorder (7A61)
Circadian rhythm sleep disorder, irregular sleep wake type (G47.23)	Irregular sleep-wake rhythm disorder (7A62)
Circadian rhythm sleep disorder, free running type (G47.24)	Non-24 h sleep–wake rhythm disorder (7A63)
Circadian rhythm sleep disorder, shift work type (G47.26)	Circadian rhythm sleep–wake disorder, shift work type (7A64)
Circadian rhythm sleep disorder, jet lag type (G47.25)	Circadian rhythm sleep–wake disorder, jet lag type (7A65)
Nonorganic sleep disorder, unspecified (F51.9) Sleep disorder, unspecified (G47.9)	Circadian rhythm sleep–wake disorder, unspecified (7A6Z)
–	*Parasomnia disorders*
Sleepwalking (somnambulism) (F51.3) Sleep terrors (night terrors) (F51.4)	Disorders of arousal from non-REM sleep (7B00)
Nightmares (F51.5)	Parasomnias related to REM sleep (7B01)
Other nonorganic sleep disorders (F51.8)	Other parasomnias (7B02)
Nonorganic sleep disorder, unspecified (F51.9) Sleep disorder, unspecified (G47.9)	Parasomnia disorders, unspecified (7B0Z)

## Insomnia disorders

Depending on the duration of insomnia symptoms, either chronic insomnia (7A00; ≥ 3 months) or short-term insomnia (7A01; < 3 months) can be diagnosed according to ICD-11. By contrast, ICD-10 does not distinguish between chronic and short-term insomnia, and a minimum duration of 1 month is required for the diagnosis of nonorganic insomnia. Regarding the frequency of insomnia symptoms, ICD-11 uses the criterion “several times per week,” whereas ICD-10 requires symptoms to occur “at least three times per week.” Accordingly, more individuals would be expected to meet this criterion under ICD-11 than under ICD-10. The criterion “poor sleep quality,” which can lead to an ICD-10 diagnosis of nonorganic insomnia even in the absence of difficulties falling or staying asleep, is no longer used in ICD-11. This is based on studies suggesting that poor sleep quality alone is clinically distinct from sleep onset or sleep maintenance difficulties [8]. ICD-11 requires that insomnia symptoms occur despite adequate opportunity and circumstances for sleep—similar to how the disorder is defined in DSM‑5. Additionally, it is noted descriptively that chronic insomnia can follow an episodic course, which is consistent with findings from natural history studies [4].

With respect to the question of whether insomnia should be diagnosed when comorbid physical illnesses or mental disorders are present, ICD-11 introduces a substantial change compared to ICD-10. According to ICD-10, the diagnosis of nonorganic insomnia should only be made in the absence of potentially underlying physical illnesses or mental disorders. By contrast, ICD 11 allows for the diagnosis of insomnia disorder whenever it represents a focus of independent clinical attention. This change acknowledges that it is often difficult for clinicians to determine whether a physical illness or mental disorder is truly the cause of insomnia symptoms. In addition, the change emphasizes that insomnia disorder can warrant independent clinical attention even when a comorbid condition is present. Accordingly, newer clinical guidelines include recommendations for the treatment of comorbid insomnia [5, 6].

## Hypersomnolence disorders

The ICD-10 diagnosis “non-organic hypersomnia” is no longer used in ICD-11. Instead, the diagnosis “hypersomnia associated with a mental disorder” (7A25) may be assigned when a mental disorder is present and the hypersomnia symptoms are of sufficient severity to warrant independent clinical attention. In ICD-10, “non-organic hypersomnia” did not necessarily require the presence of a comorbid mental disorder, making it clinically and conceptually difficult to distinguish from idiopathic hypersomnia. Consequently, many patients previously diagnosed with “non-organic hypersomnia” but without a comorbid mental disorder may now be classified under “idiopathic hypersomnia” (7A21) in ICD-11. In ICD-11, the minimum duration required for the diagnosis “idiopathic hypersomnia” is specified as “several months,” whereas the corresponding criterion in ICD-10 required a minimum duration of 1 month. Additionally, the diagnostic criteria for idiopathic hypersomnia have been further refined in ICD-11. Specifically, an average sleep onset latency of ≤ 8 min in the Multiple Sleep Latency Test (MSLT) and the absence of sleep-onset REMs in the MSLT are required. These criteria align with those of the International Classification of Sleep Disorders (ICSD‑3; [1]). ICD-11 includes the diagnoses “hypersomnia due to a medical condition” (7A23) and “hypersomnia due to a medication or substance” (7A24). These diagnoses should only be used when the hypersomnia symptoms are of such severity that they represent a primary focus of clinical attention. Kleine–Levin syndrome, previously classified as “other sleep disorder” in ICD-10, is recognized as an independent diagnostic entity in ICD-11 (7A22). Similarly, insufficient sleep syndrome, formerly included under “other nonorganic sleep disorders” in ICD-10, has been newly introduced as a separate diagnosis in ICD-11 (7A26).

## Circadian rhythm sleep–wake disorders

With regard to circadian rhythm sleep–wake disorders, the changes from ICD-10 to ICD-11 are relatively minor. The relevant ICD-10 diagnoses in Chap. V “Mental and Behavioural Disorders” (nonorganic disorder of the sleep–wake schedule, F51.2) and Chap. VI “Diseases of the Nervous System” (G47.2-) were combined in ICD-11. However, the specific ICD-11 diagnoses largely correspond to ICD-10 codes G47.21–G47.26. The wording of the ICD-11 diagnoses has been refined to specify that the symptoms must persist for several months and cause significant distress or impairment in psychological, physical, social, occupational, or educational functioning in order to justify a diagnosis.

## Parasomnia disorders

In addition to sleepwalking (somnambulism; 7B00.1) and sleep terrors (night terrors; 7B00.2), ICD-11 makes it possible, for the first time, to diagnose confusional arousals (7B00.0) and sleep-related eating disorder (7B00.3). Confusional arousals are characterized by mental confusion or confused behavior during a partial arousal from deep sleep. Sleep-related eating disorder is characterized by recurrent episodes of involuntary excessive or dangerous eating or drinking that occur during the main sleep period. All of these diagnoses fall under the umbrella term of disorders of arousal from non-REM sleep. Parasomnias related to REM sleep include REM sleep behavior disorder (7B01.0), recurrent isolated sleep paralysis (7B01.1), and nightmare disorder (7B01.2). All of these diagnoses were already recognized as diagnostic categories in ICD-10. Furthermore, ICD-11 now enables the diagnoses of hypnagogic exploding head syndrome (see [7]), sleep-related hallucinations, and parasomnias due to a medical condition or substance as “other parasomnias.” As with circadian rhythm sleep–wake disorders, parasomnias should only be diagnosed when the symptoms are severe enough to cause significant distress or substantial impairment in personal, familial, social, educational, occupational, or other important areas of functioning, or when they pose a meaningful risk of injury to the individual or others.

## Practical conclusion

- The most important structural change from ICD-10 to ICD-11 is the introduction of an individual chapter titled “Sleep–Wake Disorders,” which includes all sleep disorders.
- As a result of this, the distinction between organic and nonorganic sleep disorders has been eliminated in ICD-11. This change is justified by the clinical observation that making a valid differentiation between these categories is rarely feasible in routine practice.
- Insomnia disorder can now be diagnosed comorbidly with other mental disorders or physical illnesses. Moreover, nonrestorative sleep as the sole nocturnal symptom is no longer sufficient for the diagnosis of insomnia disorder.
- Several new diagnoses have been introduced in ICD-11. These include insufficient sleep syndrome, confusional arousals, sleep-related eating disorder, and the hypnagogic exploding head syndrome.

## Declarations

This article does not contain any studies with human participants or animals performed by any of the authors.

The supplement containing this article is not sponsored by industry.
